# Utilization of lignocellulosic biofuel conversion residue by diverse microorganisms

**DOI:** 10.1186/s13068-022-02168-0

**Published:** 2022-06-24

**Authors:** Caryn S. Wadler, John F. Wolters, Nathaniel W. Fortney, Kurt O. Throckmorton, Yaoping Zhang, Caroline R. Miller, Rachel M. Schneider, Evelyn Wendt-Pienkowski, Cameron R. Currie, Timothy J. Donohue, Daniel R. Noguera, Chris Todd Hittinger, Michael G. Thomas

**Affiliations:** 1 Department of Bacteriology, University of Wisconsin-Madison, 1550 Linden Dr, Madison, WI 53706 USA; 2 Wisconsin Energy Institute, University of Wisconsin-Madison, 1552 University Ave, Madison, WI 53726 USA; 3 Laboratory of Genetics, Center for Genomic Science Innovation, J. F. Crow Institute for the Study of Evolution, University of Wisconsin-Madison, 425-g Henry Mall, Madison, WI 53706 USA; 4 Department of Civil and Environmental Engineering, University of Wisconsin-Madison, 1415 Engineering Dr, Madison, WI 53706 USA; 5 DOE Great Lakes Bioenergy Research Center, University of Wisconsin-Madison, 1552 University Ave, Madison, WI 53726 USA

**Keywords:** Biofuel, Conversion residue, Lignocellulose, *Streptomyces*, Valorization, Yeasts

## Abstract

**Background:**

Lignocellulosic conversion residue (LCR) is the material remaining after deconstructed lignocellulosic biomass is subjected to microbial fermentation and treated to remove the biofuel. Technoeconomic analyses of biofuel refineries have shown that further microbial processing of this LCR into other bioproducts may help offset the costs of biofuel generation. Identifying organisms able to metabolize LCR is an important first step for harnessing the full chemical and economic potential of this material. In this study, we investigated the aerobic LCR utilization capabilities of 71 *Streptomyces* and 163 yeast species that could be engineered to produce valuable bioproducts. The LCR utilization by these individual microbes was compared to that of an aerobic mixed microbial consortium derived from a wastewater treatment plant as representative of a consortium with the highest potential for degrading the LCR components and a source of genetic material for future engineering efforts.

**Results:**

We analyzed several batches of a model LCR by chemical oxygen demand (COD) and chromatography-based assays and determined that the major components of LCR were oligomeric and monomeric sugars and other organic compounds. Many of the *Streptomyces* and yeast species tested were able to grow in LCR, with some individual microbes capable of utilizing over 40% of the soluble COD. For comparison, the maximum total soluble COD utilized by the mixed microbial consortium was about 70%. This represents an upper limit on how much of the LCR could be valorized by engineered *Streptomyces* or yeasts into bioproducts. To investigate the utilization of specific components in LCR and have a defined media for future experiments, we developed a synthetic conversion residue (SynCR) to mimic our model LCR and used it to show lignocellulose-derived inhibitors (LDIs) had little effect on the ability of the *Streptomyces* species to metabolize SynCR.

**Conclusions:**

We found that LCR is rich in carbon sources for microbial utilization and has vitamins, minerals, amino acids and other trace metabolites necessary to support growth. Testing diverse collections of *Streptomyces* and yeast species confirmed that these microorganisms were capable of growth on LCR and revealed a phylogenetic correlation between those able to best utilize LCR. Identification and quantification of the components of LCR enabled us to develop a synthetic LCR (SynCR) that will be a useful tool for examining how individual components of LCR contribute to microbial growth and as a substrate for future engineering efforts to use these microorganisms to generate valuable bioproducts.

**Supplementary Information:**

The online version contains supplementary material available at 10.1186/s13068-022-02168-0.

## Background

Biofuel generation is viewed by many as a key component of a new low-carbon fuels and chemicals supply chain. While research has focused on improving biofuel generation pipelines, technoeconomic analysis has shown that gaining value from the portion of lignocellulosic biomass not used for biofuel generation could help make advanced cellulosic biorefineries more economically viable [1]. Currently, the residue left over after the conversion of lignocellulose to biofuel is burned to power the biorefinery [2]. However, approximately half of the chemical potential energy of the original hydrolysate remains in the residue [3]. Instead of being burned, this lignocellulosic conversion residue (LCR) could be subjected to further microbial processing with the goal of generating valuable bioproducts in addition to the biofuel [1]. For example, recent technoeconomic analyses that consider the lignocellulosic biomass, biorefinery, and biofuel generation pipeline have proposed terpenes, medium-chain fatty acids, and/or bio-based or renewable replacement chemicals as promising prospects for bio-production [3–6]. LCR consists of both soluble and insoluble components that can be subjected to valorization. The soluble LCR from ethanol fermentations is a combination of partially hydrolyzed and soluble plant material, deconstruction residues, unfermented sugars, microbial waste products, and cell debris that remain after treated lignocellulosic biomass is subjected to microbial fermentation and distillation to remove the biofuel [3]. National renewable fuels requirements continue to increase with a greater demand for lignocellulose-derived fuels and alternatives to bioethanol including biobutanol and compounds that can be used as sustainable aviation fuels [7–9]. Biorefineries are working on meeting this demand by implementing biomass treatments that increase the amounts of hexoses and pentoses available to primary fermenters [9, 10] and engineering primary fermenters that can maximize conversion of those sugars to biofuels [7, 11].

Previous analyses of a *Saccharomyces cerevisiae* Y128-fermented, switchgrass-derived soluble LCR identified many components, including the most abundant treatment residue, acetamide; most abundant sugar, xylose; and lignocellulose-derived aromatics that included known lignocellulosic-derived inhibitors (LDIs) [3]. Residual xylose levels were high in this soluble LCR due to the limited capacity of *S. cerevisiae* Y128 to ferment xylose, resulting in consumption of only 47% of the xylose contained in the hydrolysate. Since complete consumption of the xylose in the hydrolysate is a major engineering goal for primary fermentation microbes [10], it is unlikely that residual xylose will remain this high in future industrially relevant soluble LCRs. Future LCRs are expected to contain a wide range of organic carbon sources, any combination of which could serve as substrates for microbial growth. It is very possible that these LCRs will also contain inhibitors to microbial growth, such as LDIs or other residues remaining from the breakdown of plant biomass and bacterial waste products [11–13].

Given the wide range of both potential substrates and inhibitors predicted to be present in LCR and the goal of downstream engineering to generate bioproducts, we analyzed two collections of microorganisms, *Streptomyces* (Actinobacteria) and yeasts (Ascomycota), for their ability to metabolize soluble LCR aerobically. For simplicity, henceforth LCR will refer to only soluble LCR. Our goal was to identify those strains that were capable of consuming as much of the organic substrates in LCR as possible and would therefore be best suited as chassis for genetic engineering towards the production of valuable bioproducts. Both *Streptomyces* and yeasts can grow on a wide range of carbon sources, have well-developed genetic systems for metabolic engineering, and have been used for industrial production of compounds. *Streptomyces* and many yeast species are soil-dwelling microorganisms and, as such, likely regularly consume plant biomass breakdown products [14–17]. The genetic systems developed for metabolic engineering in *Streptomyces* and those for yeasts have been shown to be portable between species and are expected to work on new strains as they are discovered [18, 19]. Industrially, *Streptomyces* are mainly used for production of antibiotics and other bioactive metabolites but can also produce various terpenes and fatty acids [20–23]. Yeasts are commonly known for industrial-scale ethanol production by *S. cerevisiae*, but several non-conventional species naturally produce or have been engineered to produce a wide variety of compounds of biotechnological interest, including long chain fatty acids, alkanes, sophorolipids, and terpenes [24–26]. The *Streptomyces* collection that we tested comprised natural isolates collected from geographically diverse sites across North America and Hawaii [27]. The yeast species tested were taxonomic type strains of a diverse sampling of species across the yeast phylogeny from the Y1000+ Project collection [28]. We hypothesized that some of these microorganisms would be more resistant to potential inhibitors and possibly better at metabolizing LCR than lab-conditioned or model species, such as *S. cerevisiae*, which specialize in rapid glucose consumption to the exclusion of other metabolites.

We also examined an aerobic mixed microbial consortium (MMC) derived from activated sludge collected at a wastewater treatment plant for LCR utilization. The benefits of examining this MMC are twofold. Firstly, we hypothesized that the synergistic relationships between the different members of a microbial consortium that has been enriched on the complex organic substrates present in domestic wastewater allow them to collectively metabolize as much of the LCR as possible. The extent of LCR consumption by the MMC can therefore be considered an upper limit of LCR biodegradability, and thus, of the potential of LCR to serve as an organic carbon source to make desired products. Secondly, if the MMC is able to utilize more of the LCR than any of the individual *Streptomyces* or yeast strains, it can serve as a reservoir for genetic material to engineer strains for increased LCR utilization.

Together, these experiments examining LCR utilization by *Streptomyces* and yeast species will identify which species can serve as the best chassis for engineering efforts to produce valuable bioproducts from LCR. Analysis of the MMC will suggest ways to engineer these microbes to utilize more LCR for conversion into valuable bioproducts and potentially provide a source of genetic material for those engineering efforts. This is an important first step in using microbial generation of products in addition to biofuels for offsetting the cost of biofuel refineries.

## Results and discussion

### Determining the composition of a model lignocellulosic conversion residue

Given that high levels of plant material deconstruction and xylose consumption are the major engineering goals for hydrolysate production and primary microbial fermentation, respectively, we aimed to characterize a LCR with minimal plant material and decreased xylose concentration. The chosen LCR was derived from an ammonia fiber expansion (AFEX)- and enzyme-treated switchgrass hydrolysate that was filtered to remove the majority of remaining insoluble plant materials [29]. This filtered hydrolysate was then subjected to fermentation by *Zymomonas mobilis* and distillation to remove bioethanol. Engineered *Z. mobilis* 2032 is known to catabolize xylose more efficiently than the similarly engineered and evolved *S. cerevisiae* Y128 [29, 30]; thus, we anticipated the remaining xylose in this LCR would be significantly reduced in comparison to the yeast-fermented LCR. Furthermore, glucose was not expected to be a major component of this LCR as it was predicted to be almost fully consumed during primary fermentation. Consistent with these hypotheses, Z. *mobilis*-based fermentation resulted in approximately 90% xylose consumption, almost complete glucose consumption, and a final LCR COD of approximately 70 g COD/L (compared to about 146 g COD/L of the hydrolysate) [29] (Additional file 1: Table S1).

**Fig. 1:**
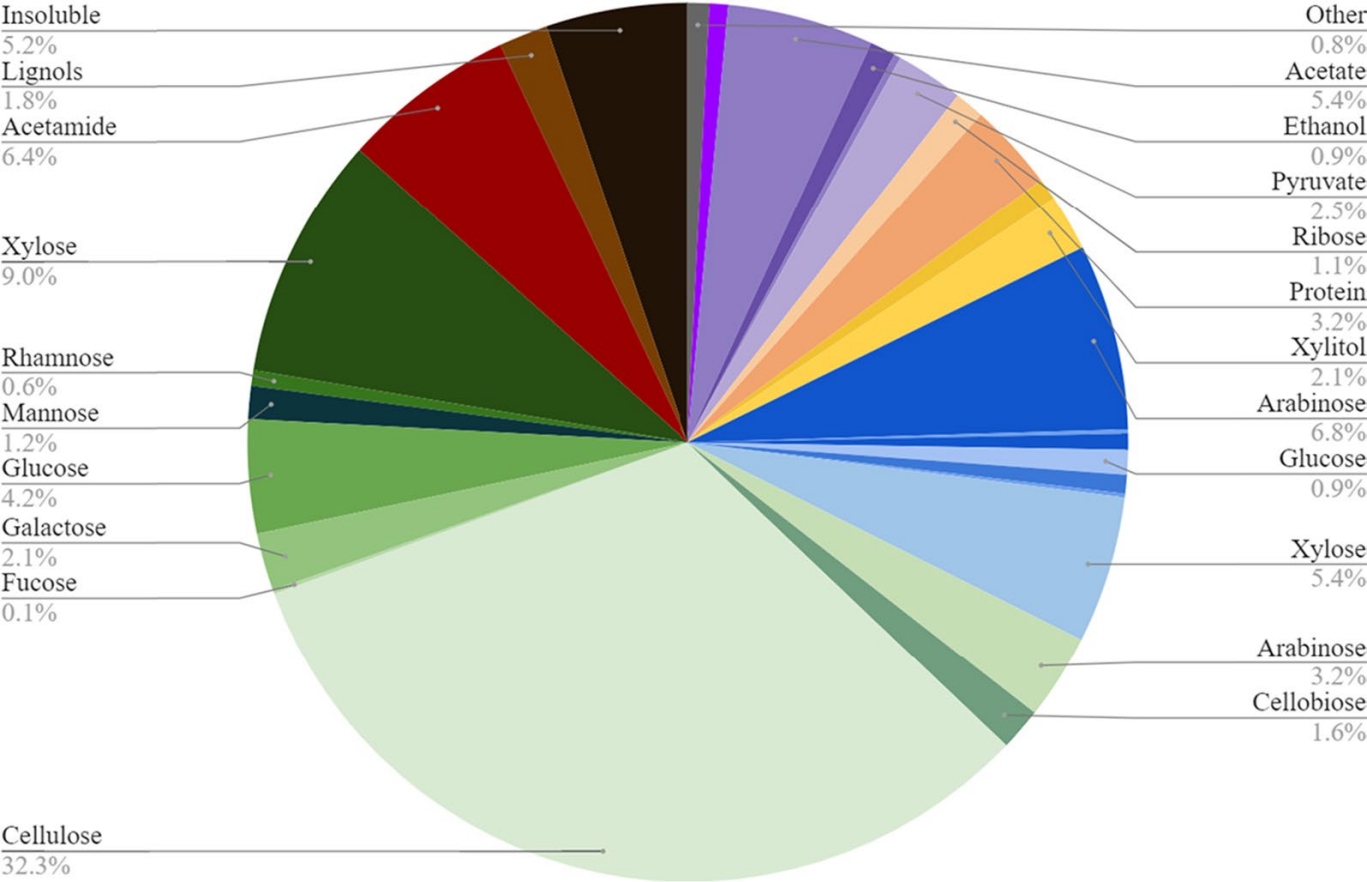
Composition of lignocellulosic conversion residue. Lignocellulosic conversion residue (LCR) was generated from “Cave in Rock” switchgrass harvested in 2016, subjected to AFEX and enzyme pretreatments, fermented by *Zymomonas mobilis*, and finally distilled to remove ethanol. The total potential chemical energy of the remaining LCR was determined by COD analysis while the remaining carbon sources, *Zymomonas* waste products, and other compounds that might affect future microbial metabolism were quantified via a combination of different separation techniques combined with HPLC and GC–MS analysis then converted to g/L COD to calculate the percent composition of the LCR. The most abundant components of this LCR were oligomeric sugars shown in shades of green (54.2%), monomeric sugars in shades of blue (14.7%), C1–C4 metabolites in shades of purple (10.8%) and acetamide in red (6.4%). These numbers are the average of five batches of LCR

To better understand the composition of this model LCR, a combination of HPLC- and GC–MS-based assays were employed to analyze several batches of this LCR. These analyses identified and quantified the amounts of sugars and other metabolites present that may be used as substrates for further fermentation to additional bioproducts (Fig. 1, Additional file 1: Table S1). This LCR was mostly liquid with about 0.7% solids by weight. The largest component of the COD of LCR was oligomeric sugars (54.2 ± 1.8%). The monomeric sugars were the next largest component at 15% of the total COD and included mostly arabinose (6.8 ± 0.2%) and xylose (5.4 ± 0.8%), with trace amounts of glucose, galactose, mannose, fucose, and rhamnose. Metabolites containing up to four carbons (C1–C4) comprised about 11% of the total COD and included primarily acetate (5.4 ± 0.1%), pyruvate (2.5 ± 0.3%), and several other organic compounds. Acetamide, the main residue produced during the nitrogen-rich AFEX pretreatment [31], was one of the largest single components of the LCR other than sugars (ca. 6%). The alcohols glycerol and xylitol made up a very small percent of the total COD of LCR, 2.6 ± 0.9%. Other compounds present in the LCR could be inhibitory to cell growth, such as monolignols from the plant matter (1.8 ± 0.2%) [11–13, 32]. The remaining insoluble fraction of the COD (ca. 5%) is most likely residual cell debris from the hydrolysate-fermenting microorganisms (e.g., *Zymomonas*). Any remaining material and any leftover COD in the form of amino acids or sugars not accounted for previously were designated as ‘other’ (ca. 0.8%).

### Growth of microorganisms on model lignocellulosic conversion residue

We tested 71 *Streptomyces* and 163 yeast species aerobically in batch cultures grown at standard growth temperatures until they reached stationary phase to identify strains in these two groups of organisms that grew well in the model LCR. For comparison, activated sludge from a wastewater treatment plant was also fed with the model LCR and samples taken were grown aerobically in batch culture for 7 days at room temperature. Growth on LCR was measured as the average dry cell weight (DCW) of several biological replicates.

**Fig. 2:**
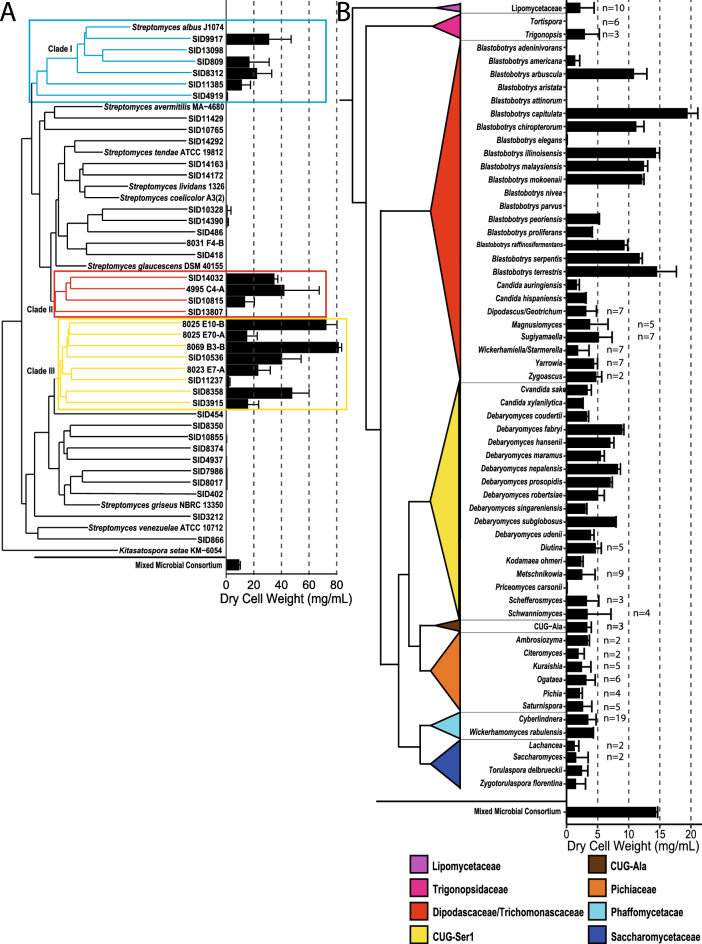
Phylogenetic trees and growth on lignocellulosic conversion residue. Phylogenetic trees of select *Streptomyces* (**A**) and yeast species (**B**) show the diversity within the tested strains. The bar graphs depict growth of these microorganisms in LCR as the average dry cell weight (mg/mL) of at least 2 mLs of culture from at least two biological replicates with the average dry cell weight (mg/mL) of the microbial consortium at the bottom of each panel. *Streptomyces* strains capable of moderate (≥ 5 mg/mL DCW) and high (≥ 10 mg/mL DCW) growth after seven days at 28 °C with shaking formed distinct phylogenetic groupings indicated as clade 1 (blue), 2 (red), and 3 (yellow) above. Similarly, the highest growing yeast species after four days rolling at room temperature were from two distinct clades: the Dipodascaceae/Trichomonascaceae clade containing the *Blastobotrys* or the CUG-Ser1 clade containing the *Debaryomyces*. The number (n) of species in condensed yeast clades is indicated, and the reported values are the mean and standard deviation of values for all species in that clade. Full growth data are available in Additional file 2: Table S2. Clade, species, and strain designations are available in Additional file 5: Table S7

Of the 71 *Streptomyces* strains tested, more than half were able to grow in LCR. Of these, 28 had at least moderate growth (≥ 5 mg/mL DCW) and 22 of those had high growth of ≥ 10 mg/mL DCW (Additional file 2: Table S2). A phylogenetic analysis of a subset of these *Streptomyces* strains showed that strains capable of growing to high DCW form distinct phylogenetic groupings with the majority of strains capable of growth on LCR falling into 3 clades, suggesting that there might be multiple factors that contribute to the ability of *Streptomyces* to grow on LCR (Fig. 2A). While each of these three clades contained at least one strain capable of high growth, the majority of high-growth strains occurred in clade III. A sizable fraction of yeast species, 34 out of 163, showed moderate growth on the model LCR (Fig. 2B). Some species, including *S. cerevisiae*, were likely unable to grow in LCR due to the low concentration of glucose, their preferred carbon source [33], confirming our hypothesis that an appetite for diverse carbon sources is vital to yeast growth in LCR. Other species, such as *Lipomyces starkeyi*, are known to consume xylose and other diverse substrates [34], so the lack of growth is likely due to factors beyond carbon source availability. Several species in the same family, Lipomycetaceae, grew in LCR which suggests that essential traits are variable among close relatives. The highest growing yeast species fell into two distinct groups; several of these yeasts were in the Dipodascaceae/Trichomonascaceae clade, particularly in the genus *Blastobotrys,* and the rest were in the CUG-Ser1 clade, particularly in the genus *Debaryomyces* (major clade terminology based on [35])*.* Both of these genera contain yeasts with traits of biotechnological interest, including lipid production capacity in some *Blastobotrys* yeasts, food applications of *Debaryomyces* species, and tolerance by both groups to an array of stressors [36].

The *Streptomyces* species capable of high growth had a significantly higher maximum DCW than the tested yeasts, with 8069 B3-B at 81.5 ± 2.1 mg/mL and *Blastobotrys capitulata* at 19.6 ± 1.6 mg/mL. For comparison, growth yield from the microbial consortium was high, at approximately 14 mg/mL DCW. Although this was lower than several of the *Streptomyces* strains, the growth yield was comparable to the highest growing *Blastobotrys* yeasts.

### Utilization of lignocellulosic conversion residue components

**Fig. 3:**
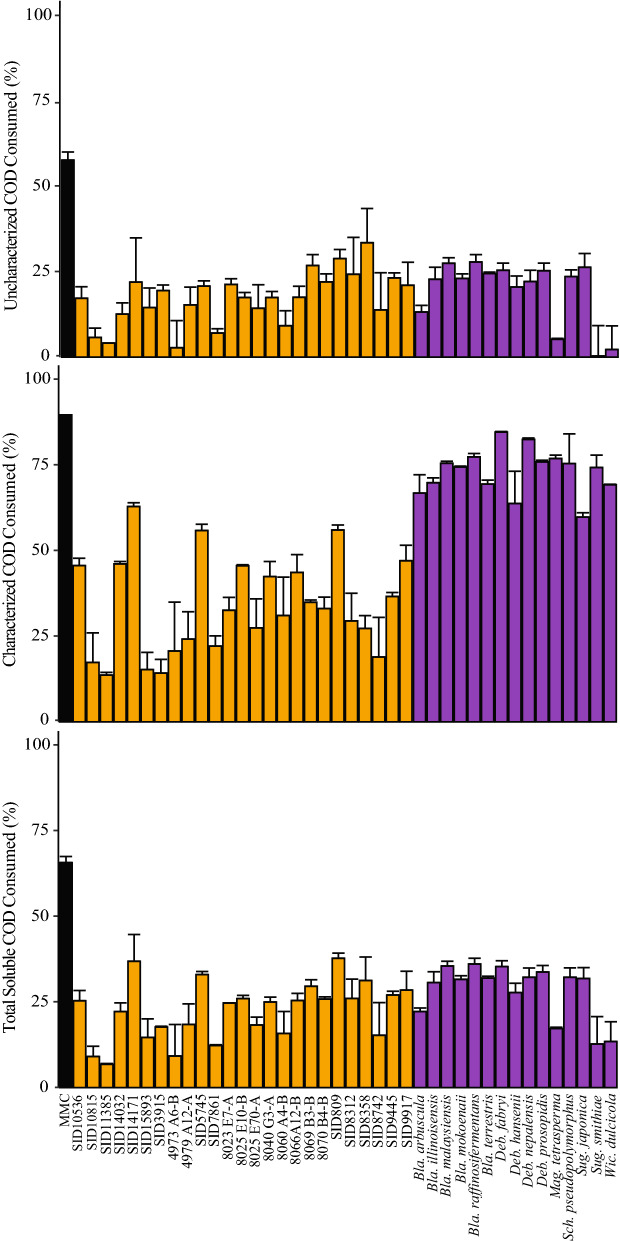
Utilization of lignocellulosic conversion residue by *Streptomyces*, yeasts, and mixed microbial consortium. Microbes were incubated in LCR then subjected to COD assays and metabolite analyses via HPLC. *Streptomyces* are shown in orange, yeasts in purple, and the mixed microbial consortium (MMC) in black on each panel. Percent of soluble COD utilized after incubation of indicated microbes in LCR is calculated relative to a media control. Characterized metabolites include C1–C6 compounds formate, acetate, ethanol, succinate, pyruvate, propionate, lactate, glycerol, xylitol, xylose, and glucose, as well as the glucose dimer cellobiose. The uncharacterized fraction includes all other soluble components such as oligomeric sugars, monolignols from the plant matter, AFEX pretreatment residues, cell debris, or other metabolic byproducts. Values reported are the average of at least two biological replicates with standard deviation denoted by error bars

While COD gives a measurement of potential chemical energy contained in the tested media, we wanted to perform a more detailed analysis on which carbon and energy sources the *Streptomyces* and selected yeasts were using for growth. All *Streptomyces* that grew in LCR, the highest growing yeast species of the *Blastobotrys* and *Debaryomyces* genera, and some related species were chosen for further characterization. We quantified the amounts of sugar alcohols, glucose, xylose, cellobiose, pyruvate, succinate, lactate, formate, acetate, and ethanol in LCR before and after growth from the previous experiment. The sum of COD concentrations of these components was reported as characterized COD (Fig. 3, Additional file 2: Table S2), and all other LCR components (i.e., oligomeric carbohydrates, monolignols from the plant matter, AFEX pretreatment residues, cell debris, or other metabolic byproducts) were reported as uncharacterized COD (Fig. 3, Additional file 2: Table S2). Individual *Streptomyces* strains were capable of consuming up to 62.9 ± 1.1% of the characterized COD (i.e., SID14171, 14.6 ± 0.3 g COD/L) and 33.4 ± 10.0% of the uncharacterized COD (i.e., SID8358, 13.9 ± 4.2 g COD/L), and a maximum of 37.7 ± 1.5% of the total soluble COD (i.e., SID809, 25.4 ± 1.0 g COD/L) (Fig. 3, Additional file 2: Table S2). Yeast strains consumed a similar amount of the overall COD; 36.1 ± 1.7% of the total soluble COD (e.g., *Blastobotrys raffinosifermentans*, 21.8 ± 1.0 g COD/L), but generally consumed a greater portion of the characterized components (e.g., *Debaryomyces fabryi*, 82.3 ± 0.2%, 8.3 ± 0.0 g COD/L) than *Streptomyces* (Fig. 3, Additional file 2: Table S2). Most individual microbes consumed a larger percentage of the characterized components as compared to the uncharacterized components. *Streptomyces* strains SID3915 and SID8358 were exceptions to this pattern, consuming a higher percentage of the uncharacterized COD (19.4 ± 1.7%, 8.8 ± 0.7 g COD/L; 33.4 ± 10.0%, 13.9 ± 4.2 g COD/L, respectively) than the characterized COD (14.2 ± 40%, 3.2 ± 0.9 g COD/L; 27.3 ± 3.7%, 6.1 ± 0.8 g COD/L, respectively), suggesting that these strains had a preference for the components in the uncharacterized portion of the LCR or were perhaps better at accessing those components than the other strains tested (Additional file 2: Table S2). For comparison, the MMC consumed the highest percent of the soluble COD at 65.7 ± 1.9% (40.4 ± 1.2 g COD/L), nearly 90% (ca. 14 g COD/L) of the characterized substrates comprising LCR, and almost 60% (ca. 26 g COD/L) of the uncharacterized components (Fig. 3, Additional file 2: Table S2). This consumption was approximately 25% higher overall than any individual microbe, mostly through consumption of the uncharacterized material. It was surprising that although *Streptomyces* strains had the highest biomass accumulation as indicated by DCW, they were not the highest consumers of LCR COD. The microbial consortium likely had a higher rate of respiration than any individual species tested which would explain the comparatively low biomass for the amount of COD consumption. Although the MMC consumed a majority of the COD in LCR, a large concentration of soluble organic compounds (both characterized and uncharacterized components) was still present following a 7-day incubation, ca. 20 g COD/L. This suggests that a portion of the LCR COD may be inaccessible to this consortium and may be entirely inaccessible to biofuel- and bioproduct-producing microbes, such as *Zymomonas*, *Streptomyces* and yeasts. Reducing the residual fraction of COD is a potential target for further improvements of the upstream processing of biomass prior to primary fermentation.

**Fig. 4:**
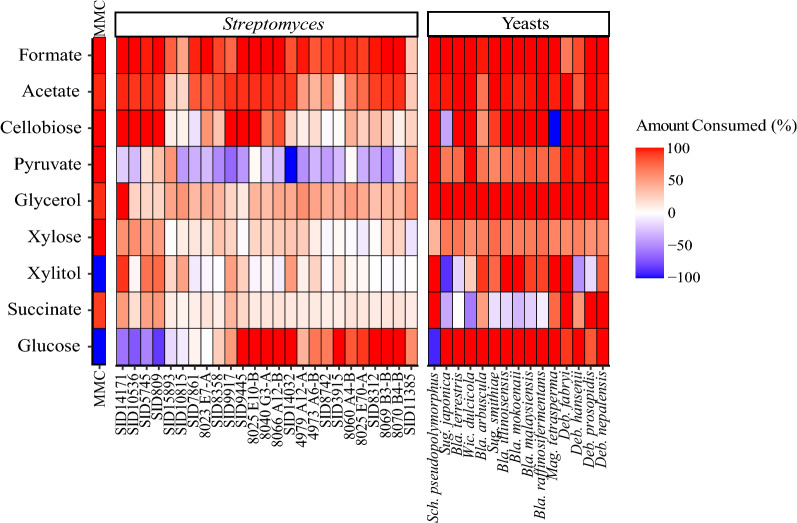
Lignocellulosic conversion residue metabolite utilization by *Streptomyces*, yeasts, and mixed microbial consortium. Patterns of indicated characterized metabolites present in LCR after incubation with microbes are shown relative to media controls. Analysis of at least two biological replicates was averaged. Metabolites that were present in lower levels than the media control are shown in red, metabolites that were present in higher levels than the media control are shown in blue, and white indicates no change relative to the media control. This gives a pattern of characterized metabolite consumption (red) and generation (blue) for these LCR degrading microbes

HPLC analysis of the characterized components of LCR after microbial growth focused on three groups of compounds: C1–C4 metabolites, monomeric sugars, and sugar alcohols (Fig. 4). One limitation of this HPLC analysis is that xylose and galactose, both known components of LCR, eluted from the column at the same time. Since galactose is only present in small amounts in LCR (Additional file 1: Table S1), we will refer to this combined value as xylose going forward.

As expected, the levels of many of the assayed compounds were lower after incubation with these microorganisms as they were energy sources for cell growth. However, some of the spent LCR samples tested showed increased levels of pyruvate, succinate, xylitol, xylose, cellobiose, and/or glucose after microbial growth. (Fig. 4) The majority of *Streptomyces* strains produced high amounts of pyruvate, a known behavior of *Streptomyces* growing in media with high nitrate concentrations [37]. Three of the yeast species produced noticeable levels of succinate, a known anaerobic byproduct of some yeasts that is hypothesized to be driven by membrane energization, which may suggest that even though the cultures were grown with rolling, oxygen was limiting under these growth conditions [38].

Since xylose, cellobiose, and glucose are rare extracellular products for microorganisms, they were most likely being generated as breakdown products from the solubilized cellulose (cellobiose, glucose) and hemicellulose (xylose) dimers or small polymers remaining from hydrolysis of the original plant material and that were not metabolized during primary fermentation. This hypothesis is consistent with the utilization of uncharacterized COD depicted in Fig. 3. The abundance of these sugars after microbial growth is likely due either to their production at a rate higher than their uptake or whose uptake was prevented by catabolite repression. Furthermore, the patterns of metabolite consumption could indicate a preference for cellulose over hemicellulose by *Streptomyces*, as many strains show higher consumption of glucose and cellobiose than xylose and xylitol. However, the apparent accumulation of glucose in spent LCR from the MMC and several *Streptomyces* strains was surprising, as glucose is a preferred carbon source for many microorganisms, and we hypothesized that the consortium would utilize all available sugars. Further examination of the HPLC traces suggest that another byproduct from these samples eluted from the HPLC at approximately the same time as glucose (Additional file 1: Figure S1).

From these growth and LCR consumption assays, we can identify microorganisms that are good candidate chassis for generating valuable bioproducts from LCR. A high overall soluble COD consumption indicates that more energy would be available to be pushed towards the production of these compounds. As microbes are engineered for higher COD consumption, we hypothesize that it will be easier to increase consumption of the characterized LCR components than the uncharacterized. This would make microbes that already have high consumption of the uncharacterized portion of the LCR more desirable production chassis. For the *Streptomyces*, SID14171 and SID809 perform well in overall COD consumption, but SID8358 is perhaps a more attractive target due to its superior performance on the uncharacterized portion of the LCR. For the yeast species, performance on the characterized portion of COD was strong for all species analyzed. Generally, performance was also similar across species on the uncharacterized portion, but several species including *Sugiyamaella smithiae*, *Wickerhamiella dulciola*, and *Blastobotrys arbuscula* proved weaker in this regard. *Blastobotrys raffinosifermentans* has recently been highlighted due to its potential for lipid production and may prove to be a strong chassis for bioproduct formation from LCR [39].

### Development of synthetic conversion residue

Given the complexity and limited availability of LCR, we wanted to develop a defined media that mimicked LCR for future experiments. This synthetic conversion residue (SynCR) would allow us to examine how individual components affect the growth of our microorganisms and if any uncharacterized components have a relevant contribution to growth phenotypes. To design SynCR, we used the analysis of the LCR as a starting point. We included the sugar alcohols, C1–C4 metabolites, cellular waste products, and the most abundant monomeric sugars. Since it was not possible to determine the chain length of the oligomeric sugars and adding variable chain-length purified hemicellulose or cellulose consistent with our observed compositions was not logistically feasible, we added the most abundant hemicellulose components as monomers and used Sigmacell50 to represent the cellulose. Cysteine, methionine, and tryptophan were not detected in our analyses, but they were added to the SynCR at 150 μM to ensure growth and optimize future production of bioproducts [40, 41]. The remaining amino acids were added at the concentrations measured in LCR. The most abundant minerals and metals (≥ 3 mg/mL), as well as any required for growth by either yeasts or *Streptomyces*, were also included in the final SynCR. Acetamide was included in SynCR as it is a significant component of LCR. After the addition of all these components except for the Sigmacell50, the SynCR was filtered and adjusted to pH 6.5, the same pH as LCR for microbial growth. The Sigmacell50 was sterilized by autoclaving and added to the SynCR after final filtration (recipe in Additional file 1: Table S3).

**Fig. 5:**
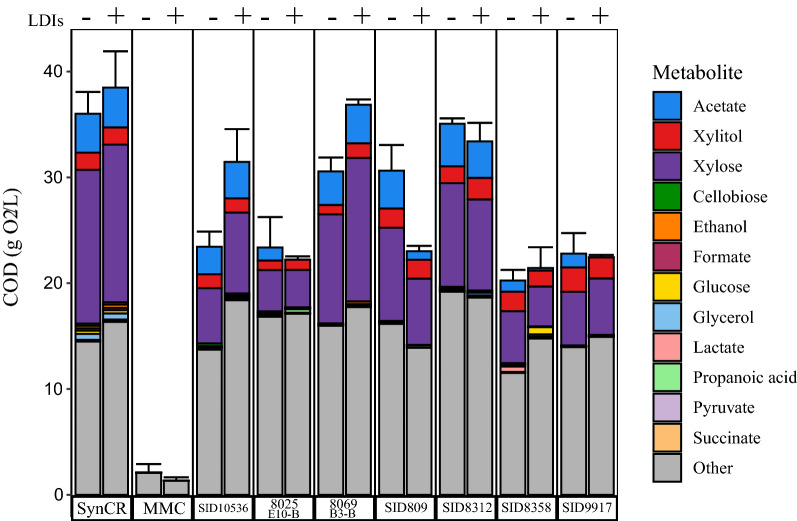
Utilization of synthetic conversion residue by *Streptomyces* and mixed microbial consortium. Microbes were incubated aerobically in synthetic conversion residue (SynCR) with crystalline cellulose and with or without lignocellulose-derived inhibitors (LDIs) for seven days then subjected to COD assays and metabolite analyses via HPLC. The bars labeled SynCR show the metabolite levels in the uninoculated media controls while the remaining bars indicate the amounts of the those metabolites present in spent SynCR after 7 days of incubation with either the mixed microbial consortium (MMC) or the indicated *Streptomyces* strains. Values reported are the average amounts of metabolites remaining after incubation from 3 biological replicates

To begin investigating how microbes utilize the wide variety of carbon sources in the LCR, we used SynCR to examine the metabolite consumption patterns of a subset of *Streptomyces* and yeasts that had a range of growth and metabolite consumption patterns on LCR. Since SynCR is a minimal version of LCR with approximately the same calculated COD (ca. 65 g/L) and the hemicellulose components converted to monomers, we hypothesized that we would see a greater percentage of the SynCR utilized by our microorganisms than the LCR. That was true for most of the *Streptomyces* strains tested, where the strains utilized as much or more of the SynCR than they did the characterized components of the LCR (Fig. 5, Additional file 3: Table S5). However, 8069 B3-B had a lower overall percent of SynCR utilized as compared to LCR, suggesting that breakdown and utilization of the uncharacterized components of LCR are an important factor in metabolite utilization by this strain. The most striking difference in the metabolites characterized in SynCR after the growth of the indicated *Streptomyces* strains was a relative increase in the “other” component as compared to the uninoculated SynCR (Fig. 5). *Streptomyces* are known to produce many specialized metabolites [42] and may be converting some of the SynCR carbon into these metabolites, which would account for the increase in the “other” COD component. It also makes it unclear how much of the uncharacterized portion of LCR was new compounds produced by the microbes assayed or material initially in the LCR that was not broken down or consumed during incubation. Another notable difference in SynCR utilization as compared to LCR utilization (Fig. 3b) was the lack of cellobiose accumulation in all strains except SID8358. Since that metabolite is present due to cellulose breakdown in LCR, this result suggests that the insoluble Sigmacell50 was not as accessible to most of the *Streptomyces* tested as the soluble switchgrass-derived lignocellulose polymers in LCR. Further, the ability of SID8358 to degrade both the uncharacterized, oligomeric-containing portion of LCR and Sigmacell50 indicate that it could serve as a potential source for future mining of cellulose-degrading enzymes.

Interestingly, only one of the seven tested yeast species was able to grow in the SynCR (Additional file 1: Table S4). Since these yeasts grew in LCR, this suggested that a component of the LCR essential for yeast growth was not included in the SynCR recipe. Standard yeast synthetic media [43] contains the vitamins biotin, inositol, and the B vitamins pyridoxine and niacin. Vitamin supplementation restored growth of yeasts in SynCR (Additional file 1: Table S4) and LC–MS/MS analysis confirmed that those vitamins were present in LCR at concentrations greater than those required for growth of the tested yeast species. In the future, any LCR generated will need to be evaluated for vitamins to assess viability of yeast growth.

The MMC was also grown on SynCR to show the maximum amount of SynCR available for microbial degradation. The MMC was able to metabolize almost the entirety of the soluble SynCR (95.2 ± 1.8%, 34.1 ± 1.6 g COD/L), which was not surprising as the consortium was also able to metabolize such a large portion of the characterized components of the LCR. The composition of future SynCR recipes could also be adjusted to focus on separate carbohydrate components, e.g., arabinose versus xylose, in order to identify or engineer microorganisms better able to metabolize these components.

One of the uses for SynCR is to test how individual components affect the growth of our microorganisms. Since our microbes were able to grow in LCR, they obviously are tolerant of LDIs which had been reported in the literature to be inhibitory to some microbes [32]. We wanted to determine if LDIs had any negative impact on the growth and LCR utilization of our microbes that might be reduced by future engineering efforts. Growth experiments using SynCR both with and without LDIs allowed us to test the effect these compounds had on our organisms. Interestingly, LDIs appear to have minimal effect on the *Streptomyces* strains tested. Only two of the *Streptomyces* strains tested, SID10536 and SID809, showed differential behavior in percent SynCR consumed in the presence or absence of LDIs. The higher percent of metabolites consumed by SID10536 when grown in SynCR without LDIs suggests that LDIs may be inhibitory or inducing other pathways that shift metabolism away from consumption, while conversely the LDIs may be inducing consumption in SID809. Due to the difficulty in getting yeasts to grow on SynCR, they were not evaluated for inhibition by LDIs.

Comparisons between the most abundant OTUs in the SynCR experiments with and without LDIs showed only minimal changes in microbial abundances. This is reflected in non-metric multidimensional scaling (NMDS)-space, where the LCR consortium plots more distantly from the two SynCR consortia (Additional file 1: Fig. S2). Only one OTU, *Corynebacterium*, had a higher relative abundance in both the LDI-containing LCR and SynCR with LDIs experiment, consistent with the observation that some *Corynebacterium* species have shown tolerance to LDIs [44]. The similarity of the microbial consortia grown on SynCR with and without LDIs suggests these potential toxins have a minimal effect on the microbial consortium structure. Furthermore, the similar abundance of microbial consortium members in both SynCR incubations (Fig. 5) and the data from the *Streptomyces* species tested both suggest that the LDIs were not universally an impediment to microbial growth. This observation is counter to previous studies demonstrating the inhibitory effect of these compounds on fermentative organisms [11–13].

#### Consortium differences in model LCR compared to SynCR

The mixed microbial consortium serves as both a representative of the maximum LCR utilization possible and as a potential source of genetic material to engineer microbes for increased LCR utilization. The SynCR can be used to assist in identification of OTUs that are responsible for utilization of specific components of the LCR. For example, to identify which OTUs in the MMC contribute to mannose utilization, we can monitor the change in consortium composition when the MMC is grown in SynCR with mannose as compared to SynCR without mannose. Similarly, as SynCR consists mostly of the characterized components of LCR, comparisons between consortium composition when grown on LCR as compared to SynCR will allow us to examine which OTUs contribute to the utilization of the uncharacterized portion of the LCR. Since we predict that it will be more difficult to engineer strains to utilize this portion of the LCR, the MMC can thereby serve as a uniquely valuable resource for that genetic material.

**Fig. 6:**
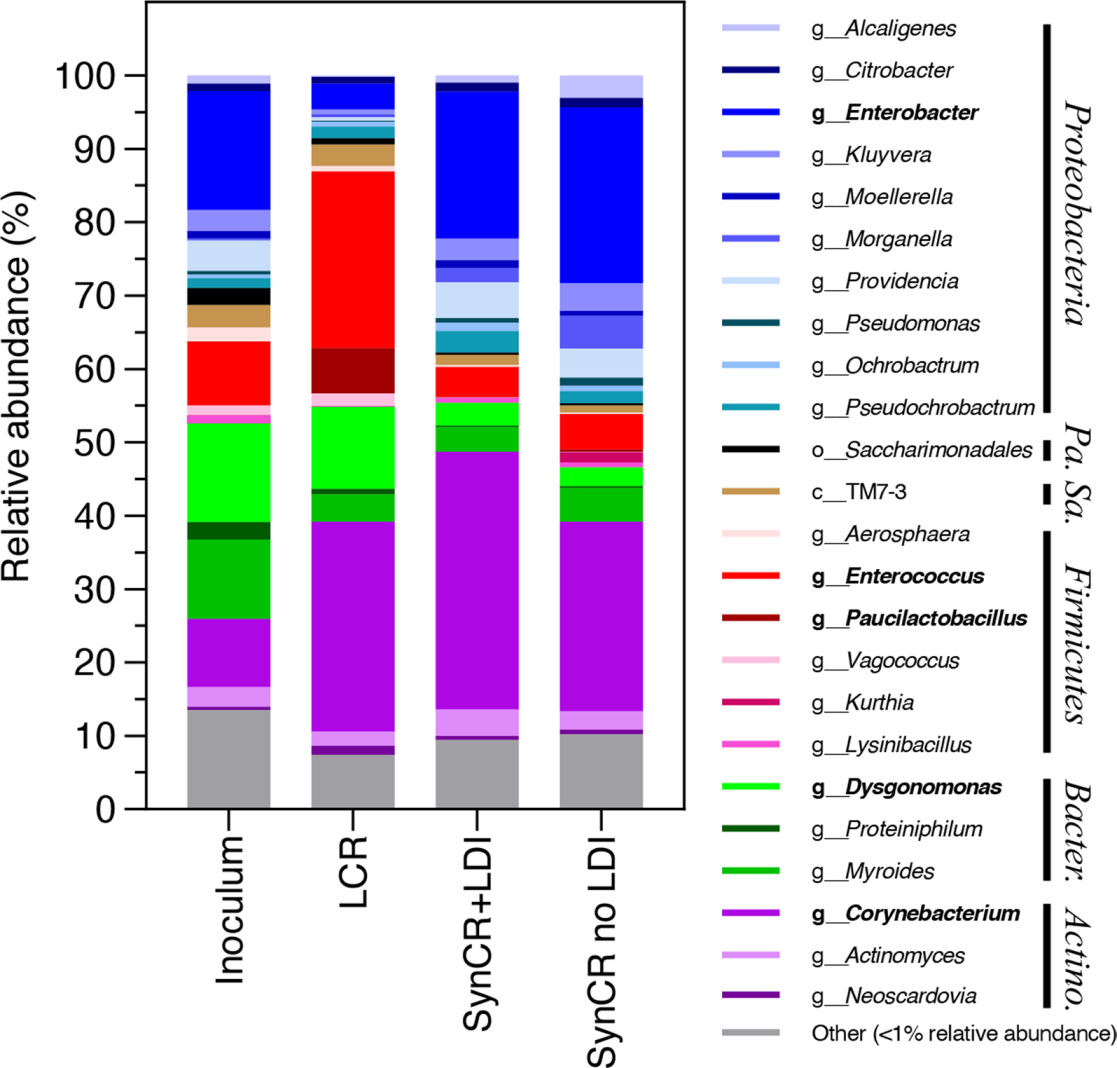
Distribution of bacterial taxa in the mixed microbial consortium on different growth media. Bacterial taxa were identified within the initial inoculum source and following a 7-day incubation period in LCR or SynCR with crystalline cellulose and with and without LDIs. Individual OTUs were clustered to the highest taxonomic level (c, class; o, order; g, genus), with clusters greater than 1% total relative abundance shown above, organized by phylum (*Pa*., *Patescibacteria*; *Sa*., *Saccharibacteria*; *Bacter*., *Bacteroidota*; *Actino*., *Actinobacteriota*). Taxa with distinct differences in abundance between the microbial consortia grown on different types of CR are indicated in bold

For these experiments, genomic DNA extracted from the initial inoculum and biomass pellets (*n* = 4) from the microbial consortia grown as indicated was subjected to 16S rRNA gene amplicon sequencing analysis. A total of 2931 unique operational taxonomic units (OTUs) were identified across all samples with the OTUs present in the initial wastewater consortium included for comparison (Additional file 4: Table S6). Subsequent analyses focused on the most abundant taxonomic groups which had a relative abundance of 1% or greater at the genus level. This procedure resulted in 24 highly abundant taxonomic groups across all samples representing approximately 90% of the total DNA reads (Fig. 6). The number of distinct highly abundant taxonomic groups in each sample tested were similar (inoculum, 17; LCR, 11; SynCR with LDIs, 15; SynCR without LDIs, 14), with a slightly less diverse microbial consortium present after seven days of growth in LCR than in SynCR, which is most likely due to less easily available carbon for microbial metabolism.

The microbial consortium analysis revealed several key differences in consortia composition when grown on LCR as compared to SynCR. The most profound difference in microbial consortium composition between the LCR and SynCR experiments was the variable abundance of *Enterococcus* and *Enterobacter*. In the LCR-fed cultures, *Enterococcus* represented approximately a quarter of the 16S rRNA sequence reads, but they were present at about 5% relative abundance in the inoculum and in both SynCR experiments. Conversely, *Enterobacter* represented 15–24% of the microbial population in the inoculum and SynCR experiments but less than 4% of the LCR consortium (Fig. 6). *Enterobacter* are endophytes, which have been associated with numerous plant species, including switchgrass [45]. These taxa have also been shown to metabolize cellulose and components of hemicellulose, such as xylose [46–48]. Species of *Enterococcus* are facultative anaerobes that are both typical commensal members of the human gut microbiome and potentially pathogenic, so their presence in these experiments is unsurprising due to the origin of the inoculum. Cellulolytic activity, as well as metabolism of other carbohydrate polymers, such as pectin, have also been reported in *Enterococcus* species [49–52], which is consistent with a typical greater abundance of these taxa in the gut microbiomes of vegetarians [53] and in fermented plant products [54]. The prevalence of lignocellulose-degrading enzymes and cellulolytic activity in *Enterobacter* and *Enterococcus* indicates a likely role that these taxa play in the LCR and SynCR consortia; however, the abundance of free monomer carbohydrates in SynCR likely allowed the *Enterobacter* to outcompete the *Enterococcus* in these experiments. Because of the apparent opportunistic behavior of the *Enterobacter*, we anticipate genomic studies of the *Enterococcus* members of the consortium may be a more promising resource for microbial engineering.

The other relevant difference between the LCR and SynCR microbial consortia was that *Dysgonomonas* and *Paucilactobacillus* were present in both consortia, but at a significantly increased relative abundance in LCR (ca. 11% and ca. 6%, respectively, Fig. 6) as compared to SynCR. *Dysgonomonas* are gut symbionts of termites and wood-boring insects, and recent genomic studies of the species have revealed an abundance of glycoside hydrolase enzymes, which suggests a role in lignocellulose degradation [55–57]. Similar to *Enterococcus*, abundance of *Dysgonomonas* in the LCR consortium is likely a result of their putative role in the degradation of oligomeric carbohydrates, which comprise half of the potential carbon energy in LCR, and is consistent with the high observed consumption of the uncharacterized portion of COD (Fig. 3). In contrast, *Paucilactobacillus* is a heterofermentative lactic acid bacteria notable for the uncommon ability to metabolize pentoses [58], so would be expected to be more abundant in SynCR experiments due to the greater concentration of monomer pentoses in the SynCR as compared to LCR. However, most of the characterized species of this genus were isolated from fermented plant material and some strains have been shown to metabolize disaccharides, such as melibiose by *Paucilactobacillus hokkaidonensis* [58], and may therefore be able to utilize some of the less commonly metabolized plant breakdown sugars. Depending on the carbohydrate composition of future LCRs, *Dysgonomonas* or *Paucilactobacillus* related members of the consortium may be a beneficial bioengineering resource for genes related to oligomeric or less commonly metabolized sugar utilization.

## Conclusions

We are interested in improving the economic potential of advanced cellulosic biorefineries by valorizing the associated carbon waste streams. Currently, these waste streams are dried and subsequently burned to generate power for the biorefinery [2]. With over half the chemical potential of the original lignocellulosic hydrolysate being contained in the soluble waste stream [3], this material has untapped potential for additional economic value through bioproduct generation [1]. The first steps towards this goal are to identify the carbon-containing components within this waste material and genetically tractable microorganisms capable of catabolizing those components. The current study determined the composition of a model conversion residue derived from AFEX- and enzyme-treated switchgrass hydrolysate that was fermented by *Z. mobilis* and distilled to remove bioethanol. The remaining carbohydrates and C1–C4 compounds in this conversion residue supported the growth of diverse collections of *Streptomyces* and yeast species and a mixed microbial consortium derived from a wastewater treatment plant. Both *Streptomyces* and yeasts are well-established as industrial producers of valuable bioproducts, suggesting these abilities can be extended to valorizing LCR. Growth assays identified several *Streptomyces* and yeast species that catabolized over a third of the total soluble COD in the LCR and could therefore serve as chassis for future bioproduct generation from LCR. We also developed a defined, synthetic conversion residue that mimics LCR to examine the effects of individual components of conversion residue on microbial growth. Using this synthetic conversion residue, we showed that LDIs, which had previously been predicted as inhibitors of microbial growth on conversion residue, actually have minimal effect on the growth of *Streptomyces* strains or the composition of the MMC. Taking this work forward, we envision the development of a series of microbial chassis that can be customized for both the catabolism of the LCR, which may vary depending on the biorefinery pipeline, and the generation of bioproducts that can be selectively produced depending on the current economic demands.

## Materials and methods

### Conversion residues

#### Model LCR

“Cave in Rock” switchgrass grown and harvested in 2016 at the Arlington Agricultural Research Center in Arlington, WI, USA was pre-treated using ammonia fiber expansion [59] and enzymatic hydrolysis. After enzymatic hydrolysis, the hydrolysate was centrifuged to remove solid materials then passed through a 0.2 μm filter to further remove solids and ensure sterility, resulting in a filter-sterilized, 7% glucan-loading hydrolysate [29]. The hydrolysate was then fermented with an engineered xylose-utilizing ethanologen, *Z. mobilis* 2032 [60] and subsequently distilled to recover ethanol, leaving conversion residue as the remaining material**.**

#### Synthetic conversion residue (SynCR)

SynCR was developed from detailed analyses on 5 batches of LCR generated as described below. SynCR contains (g/L) sodium acetate 4.585, ethanol 0.200, sodium formate 0.900, cellobiose 0.200, glucose 0.400, glycerol 0.400, sodium pyruvate 1.645, sodium succinate 0.600, xylitol 1.400, xylose 8.000, arabinose 6.000, galactose 1.500, fructose 2.340, acetamide 3.780, Sigmacell 50 24.660, KH_2_PO_4_ 2.413, CaCl_2_•6H_2_O 0.294, MgCl_2_•6H_2_O 2.584, MgSO_4_•7H_2_O 0.861, NaCl 2.342, NH_4_Cl 1.779, KCl 0.823, and (mg/L) FeSO_4_•7H_2_O 2.000, H_3_BO_3_ 15.8, CuSO_4_•5H_2_O 0.100, Na_2_MoO_4_ 0.100, MnCl_2_•4H_2_O 13.800, ZnSO_4_•7H_2_O 2.800, Ala 32.193, Arg 18.485, Asn 5.297, Asp 8.642, Cys 18.174, Gln 11.164, Glu 4.841, Gly 11.692, His 2.420, Ile 16.590, Leu 23.110, Lys 14.020, Met 22.380, Phe 58.064, Pro 18.615, Ser 10.888, Thr 11.898, Trp 30.635, Val 18.233, and Tyr 82.664. Lignocellulose-derived inhibitors (LDIs) in the SynCR are (mg/L) coumaroyl amide 166.0, feruloyl amide 132.0, syringamide 7.0, benzoic acid 46.0, coumaric acid 20.0, 4-hydroxybenzoic acid 9.0, ferulic acid 6.0, syringic acid 3.0, vanillic acid 26.0, acetovanillone 5.0, coniferyl alcohol 19.0, and 4-hydroxyacetophenone 4.0. Recipe components are detailed in Additional file 1: Table S3.

### Strains and growth conditions

Strain details are provided in Additional file 5: Table S7. *Streptomyces* used in this study were isolated, sequenced, and characterized previously [27]. For *Streptomyces* cultures, 10,000,000 spores were inoculated into 7 mLs of liquid growth media in 50 mL deep baffled flasks and grown aerobically for seven days at 28 °C with shaking at 210 rpm. Yeasts were revived on YPD medium (2% dextrose, 1% yeast extract, 2% peptone, 2% agar), passaged once through 2 mL of liquid YPD medium, then inoculated 1/200 into 2 mL of the desired growth medium for 4 days rolling at room temperature, and finally subcultured again into the same conditions. Vitamin dependency for yeasts was determined qualitatively based on growth in liquid synthetic glucose media (1 g/L complete synthetic media supplement with all amino acids, 20 g/L glucose) supplemented with vitamins at the concentrations used in yeast nitrogen base from Sigma-Aldrich. Mixed microbial consortia were pre-grown aerobically on LCR in a bioreactor inoculated with a culture derived from aerobic activated sludge from the Madison Metropolitan Sewerage District’s Nine Springs facility in Madison, WI, USA. Briefly, a 400 mL bioreactor filled with 150 mL of media was operated using a Multifors 2 parallel bench-top bioreactor system (Infors USA, Inc., Annapolis Junction, MD, USA). Temperature and pH were controlled at 22 °C and 7.0, respectively. Reactor influent and effluent pumps were operated semi-continuously (feeding at 20 min intervals) pumping a total of 25 mL of LCR into and out of the bioreactor per day, maintaining a 6-day media retention time. The bioreactor was operated for 15 days prior to inoculating individual test tubes for evaluating LCR utilization. Test tubes were prepared in duplicate for both experimental and control samples with 100 μL bioreactor contents or sterile deionized water, respectively, and 5 mL of the following three CR varieties: LCR or SynCR with and without lignocellulose-derived inhibitors (LDI). All test tubes were incubated at room temperature and grown aerobically on an orbital shaking platform at 270 rpm for 7 days.

#### Analysis of cell growth

Growth of strains was measured by dry cell weight (DCW) at time of sampling and compared to the dry cell weight of an LCR media control to account for any residual solids in the growth medium. DCW was measured after 7 days of growth for the *Streptomyces* strains and the MMC and after 4 days for the yeasts. An aliquot (2–4 mL) was taken from each culture, centrifuged, decanted, and samples were dried to evaporate residual culture medium. Dried pellets were compared to dried tubes containing the uninoculated control samples to account for any initial solid material (e.g., cell debris in the LCR), and final DCW was calculated as the difference.

### Chemical analyses

#### Chemical oxygen demand (COD) analysis

Samples (1 mL) were collected after 7 days for *Streptomyces* and microbial consortia and after 4 days for yeasts. Filtered 1/10 dilutions of those samples were subjected to COD analysis using mercury-free, High-Range COD2 Digestion Vials (Hach, Loveland, CO, USA) as per published methods [61].

#### Metabolite analysis

Samples were collected at the same time as COD analysis above, centrifuged to remove cells, then filtered and subjected to analysis by an Agilent 1260 Infinity HPLC system and refractive index detector (Agilent Technologies, Inc., Palo Alto, CA, USA). Analytes were separated using a Bio-Rad 300 × 7.8 mm Aminex HPX-87H column and Cation-H guard column (Bio-Rad, Inc., Hercules, CA, USA) at 50 °C with 0.02 N H_2_SO_4_ mobile phase and 0.5 mL min^−1^ flow rate. Levels of acetate, ethanol, formate, glucose, glycerol, lactate, pyruvate, succinate, xylitol, xylose, cellobiose, and propanoic acid were quantified as g/L in each sample.

#### Lignocellulosic conversion residue analyses

Total soluble carbohydrates (sum of monomers and oligomers) in the LCR were quantified by GC–MS following acid hydrolysis and alditol acetate derivatization [62]. Oligomeric carbohydrates in a separate set of LCR samples were quantified using the NREL Klason lignin protocol [63]. Samples were submitted to the Wisconsin State Laboratory of Hygiene for metals analysis. Briefly, LCR samples were digested using a mixture of nitric, hydrochloric, and hydrofluoric acids and hydrogen peroxide. Samples were then diluted and analyzed using a Thermo-Finnigan Element XR™ ICP-MS (Thermo Fisher Scientific, Inc., Waltham, MA, USA). Vitamin content was quantified by reverse-phase LC–MS/MS using a Waters Acquity UPLC with Quattro Micro™ API tandem mass spectrometer (Waters Corporation, Milford, MA, USA). Quantification of amino acids in LCR is described elsewhere [29] [64]. LDIs in the LCR were identified and quantified using LC–MS/MS. Samples were separated using an Acquity UPLC HSS T3 reversed phase column (150 × 2.1 mm, Waters Corp.) at 35 °C with a binary mobile phase (Phase A 0.1% acetic acid, Phase B acetonitrile) and 0.4 mL min^−1^ flow rate. The liquid chromatography system was connected to a TSQ Quantiva Triple Quadrupole mass spectrometer (Thermo Fisher Scientific, Inc.).

### Bioinformatics

Draft genomes for *Streptomyces* were generated as described before [27]. The *Streptomyces* phylogenetic tree was generated by FastTree version 2.2.0 [65] from 49 concatenated core genes that were aligned then trimmed using GBLOCKS version 1.0.4 [66, 67]. The phylogenetic tree for yeast species was built manually based on known taxonomic information and existing phylogenies [17, 35, 68]. DNA from the mixed microbial consortia was extracted from biomass pellets using the QIAGEN DNeasy^®^ PowerSoil^®^ Pro kit (QIAGEN, Inc., Germantown, MD, USA), according to manufacturer’s instructions. DNA was submitted to the University of Wisconsin Biotechnology Center (UWBC; https://www.biotech.wisc.edu/) for paired-end, 2 × 300 bp Illumina MiSeq 16S rRNA gene amplicon sequencing using primers targeting the V3–V4 region of the 16S rRNA gene [69]. Raw 16S rRNA gene amplicon sequences were processed through QIIME v1.9.1 [70], according to previously published methods [71], with the following modifications. Representative taxonomic groups from the Additional file 4: Table S6 were processed through the *summarize_taxa.py* script, which summarizes OTUs by each taxonomic level (phylum through genus).

## Supplementary Information

**Additional file 1:** **Figures S1.** and **S2.** and ** Tables S1**, **S3** and **S4**

**Additional file 2:** **Table S2.** Lignocellulosic conversion residue growth data

**Additional file 3:** **Table S5.** Synthetic Conversion Residue growth data

**Additional file 4:** **Table S6.** OTU data for the mixed microbial consortium

**Additional file 5:** **Table S7.** Strain details for *Streptomyces *and Yeasts

## Data Availability

The raw 16S rRNA gene amplicon sequencing reads are available under NCBI BioProject ID PRJNA751072.

## Abbreviations

AFEX: Ammonia fiber expansion
COD: Chemical oxygen demand
LCR: Lignocellulosic conversion residue
LDI: Lignocellulosic-derived inhibitors
MMC: Mixed microbial consortium
NMDS: Non-metric multi-dimensional scaling
OUT: Operational taxonomic unit
SynCR: Synthetic conversion residue

## References

[1] Ng RTL, Fasahati P, Huang K, Maravelias CT (2019). Utilizing stillage in the biorefinery: economic, technological and energetic analysis. Appl Energ.

[2] Gerbrandt K, Chu PL, Simmonds A, Mullins KA, MacLean HL, Griffin WM (2016). Life cycle assessment of lignocellulosic ethanol: a review of key factors and methods affecting calculated GHG emissions and energy use. Curr Opin in Biotech.

[3] Scarborough MJ, Lynch G, Dickson M, McGee M, Donohue TJ, Noguera DR (2018). Increasing the economic value of lignocellulosic stillage through medium-chain fatty acid production. Biotechnol Biofuels.

[4] Wu WZ, Maravelias CT (2018). Synthesis and techno-economic assessment of microbial-based processes for terpenes production. Biotechnol Biofuels.

[5] Wu WZ, Long MR, Zhang XL, Reed JL, Maravelias CT (2018). A framework for the identification of promising bio-based chemicals. Biotechnol Bioeng.

[6] Wu WZ, Maravelias CT (2019). Identifying the characteristics of promising renewable replacement chemicals. Iscience.

[7] Tao L, He X, Tan ECD, Zhang M, Aden A (2014). Comparative techno-economic analysis and reviews of n-butanol production from corn grain and corn stover. Biofuel Bioprod Bior.

[8] Moriarty K, Milbrandt A, Lewis J, Schwab A. 2017. Bioenergy Industry Status Report. 2017. https://www.nrel.gov/docs/fy20osti/75776.pdf.

[9] Mariano AP, Dias MOS, Junqueira TL, Cunha MP, Bonomi A, Filho RM (2013). Utilization of pentoses from sugarcane biomass: techno-economics of biogas vs. butanol production. Bioresource Technol.

[10] Parreiras LS, Breuer RJ, Avanasi Narasimhan R, Higbee AJ, la Reau A, Tremaine M (2014). Engineering and Two-Stage Evolution of a Lignocellulosic Hydrolysate-Tolerant *Saccharomyces cerevisiae* Strain for Anaerobic Fermentation of Xylose from AFEX Pretreated Corn Stover. PLoS ONE.

[11] Zhang Y, Vera JM, Xie D, Serate J, Pohlmann E, Russell JD (2019). Multiomic Fermentation Using Chemically Defined Synthetic Hydrolyzates Revealed Multiple Effects of Lignocellulose-Derived Inhibitors on Cell Physiology and Xylose Utilization in *Zymomonas mobilis*. Front Microbiol.

[12] Todhanakasem T, Yodsanga S, Sowatad A, Kanokratana P, Thanonkeo P, Champreda V (2018). Inhibition analysis of inhibitors derived from lignocellulose pretreatment on the metabolic activity of *Zymomonas mobilis* biofilm and planktonic cells and the proteomic responses. Biotechnol Bioeng.

[13] Jönsson LJ, Alriksson B, Nilvebrant NO (2013). Bioconversion of lignocellulose: inhibitors and detoxification. Biotechnol Biofuels.

[14] Schlatter DC, Kinkel LL (2014). Global biogeography of *Streptomyces* antibiotic inhibition, resistance, and resource use. FEMS Microbiol Ecol.

[15] Schlatter DC, DavelosBaines AL, Xiao K, Kinkel LL (2013). Resource use of soilborne *Streptomyces* varies with location, phylogeny, and nitrogen amendment. Microb Ecol.

[16] Opulente DA, Rollinson EJ, Bernick-Roehr C, Hulfachor AB, Rokas A, Kurzman CP (2018). Factors driving metabolic diversity in the budding yeast subphylum. BMC Biol.

[17] Kurtzman CP, Fell JW, Boekhout T, editors. The Yeasts. 5th ed. 2011.

[18] Zhao Y, Li G, Chen Y, Lu Y (2020). Challenges and advances in genome editing technologies in *Streptomyces*. Biomolecules.

[19] Lacerda MP, Oh EJ, Eckert C (2020). The model system *Saccharomyces cerevisiae* versus emerging non-model yeasts for the production of biofuels. Life.

[20] Barbuto Ferraiuolo S, Cammarota M, Schiraldi C, Restaino OF (2021). *Streptomycetes* as platform for biotechnological production processes of drugs. Appl Microbiol Biot.

[21] Martín-Sánchez L, Singh KS, Avalos M, van Wezel GP, Dickschat JS, Garbeva P (2019). Phylogenomic analyses and distribution of terpene synthases among *Streptomyces*. Beilstein J Org Chem.

[22] Kuzuyama T (2017). Biosynthetic studies on terpenoids produced by *Streptomyces*. J Antibiot.

[23] Schneider O, Ilic-Tomic T, Rückert C, Kalinowski J, Genčić MS, Živković MZ (2018). Genomics-based insights into the biosynthesis and unusually high accumulation of free fatty acids by *Streptomyces* sp. NP10. Front Microbiol.

[24] Chattopadhyay A, Mitra M, Maiti MK (2021). Recent advances in lipid metabolic engineering of oleaginous yeasts. Biotechno Adv..

[25] Buijs NA, Zhou YJ, Siewers V, Nielsen J (2015). Long-chain alkane production by the yeast *Saccharomyces cerevisiae*. Biotechnol Bioeng.

[26] Zhang Y, Nielsen J, Liu Z (2017). Engineering yeast metabolism for production of terpenoids for use as perfume ingredients, pharmaceuticals and biofuels. FEMS Yeast Res.

[27] Chevrette MG, Carlson CM, Ortega HE, Thomas C, Ananiev GE, Barns KJ (2019). The antimicrobial potential of *Streptomyces* from insect microbiomes. Nat Commun.

[28] Hittinger CT, Rokas A, Bai F-Y, Boekhout T, Gonçalves P, Jeffries TW (2015). Genomics and the making of yeast biodiversity. Curr Opin Genet Dev.

[29] Zhang Y, Serate J, Xie D, Gajbhiye S, Kulzer P, Sanford G (2020). Production of hydrolysates from unmilled AFEX-pretreated switchgrass and comparative fermentation with *Zymomonas mobilis*. Bioresour Technol.

[30] Sato TK, Tremaine M, Parreiras LS, Hebert AS, Myers KS, Higbee AJ (2016). Directed evolution reveals unexpected epistatic interactions that alter metabolic regulation and enable anaerobic xylose use by *Saccharomyces cerevisiae*. PLOS Genet.

[31] Jin M, Dale BE (2019). AFEXTM pretreatment-based biorefinery technologies. Handbook of biorefinery research and technology.

[32] Piotrowski JS, Zhang Y, Bates DM, Keating DH, Sato TK, Ong IM (2014). Death by a thousand cuts: the challenges and diverse landscape of lignocellulosic hydrolysate inhibitors. Front Microbiol.

[33] Kayikci Ö, Nielsen J (2015). Glucose repression in *Saccharomyces cerevisiae*. FEMS Yeast Res.

[34] Sitepu I, Selby T, Lin T, Zhu S, Boundy-Mills K (2014). Carbon source utilization and inhibitor tolerance of 45 oleaginous yeast species. J Ind Microbiol Biot.

[35] Shen X-X, Opulente DA, Kominek J, Zhou X, Steenwyk JL, Buh KV (2018). Tempo and mode of genome evolution in the budding yeast subphylum. Cell.

[36] Sanya DRA, Onésime D, Passoth V, Maiti MK, Chattopadhyay A, Khot MB (2021). Yeasts of the *Blastobotrys* genus are promising platform for lipid-based fuels and oleochemicals production. Appl Microbiol Biot.

[37] Madden T, Ward JM, Ison AP (1996). Organic acid excretion by *Streptomyces lividans* TK24 during growth on defined carbon and nitrogen sources. Microbiology.

[38] Duro AF, Serrano R (1981). Inhibition of succinate production during yeast fermentation by deenergization of the plasma membrane. Curr Microbiol.

[39] Thomas S, Sanya DRA, Fouchard F, Nguyen H-V, Kunze G, Neuvéglise C (2019). *Blastobotrys*
*adeninivorans* and *B*. *raffinosifermentans*, two sibling yeast species which accumulate lipids at elevated temperatures and from diverse sugars. Biotechnol Biofuels.

[40] Perli T, Wronska AK, Ortiz-Merino RA, Pronk JT, Daran J (2020). Vitamin requirements and biosynthesis in *Saccharomyces cerevisiae*. Yeast.

[41] D’Huys P-J, Lule I, van Hove S, Vercammen D, Wouters C, Bernaerts K (2011). Amino acid uptake profiling of wild type and recombinant *Streptomyces lividans* TK24 batch fermentations. J Biotechnol.

[42] Chater KF (2016). Recent advances in understanding *Streptomyces*. F1000Res.

[43] Sherman F (2002). Getting started with yeast. Methods Enzymol.

[44] Sakai S, Tsuchida Y, Okino S, Ichihashi O, Kawaguchi H, Watanabe T (2007). Effect of lignocellulose-derived inhibitors on growth of and ethanol production by growth-arrested *Corynebacterium glutamicum* R. Appl Environ Microb.

[45] Humann JL, Wildung M, Pouchnik D, Bates AA, Drew JC, Zipperer UN (2014). Complete genome of the switchgrass endophyte *Enterobacter clocace* P101. Stand Genomic Sci.

[46] Bi C, Zhang X, Ingram LO, Preston JF (2009). Genetic engineering of *Enterobacter asburiae* strain JDR-1 for efficient production of ethanol from hemicellulose hydrolysates. Appl Environ Microb.

[47] Bi C, Rice JD, Preston JF (2009). Complete fermentation of xylose and methylglucuronoxylose derived from methylglucuronoxylan by *Enterobacter asburiae* strain JDR-1. Appl Environ Microbiol.

[48] Ren Y, Wang J, Liu Z, Ren Y, Li G (2009). Hydrogen production from the monomeric sugars hydrolyzed from hemicellulose by *Enterobacter aerogenes*. Renew Energ.

[49] Ramsey M, Hartke A, Huycke M. Enterococci: From Commensals to Leading Causes of Drug Resistant Infection. 2014. 24649510

[50] Hoheneder R, Fitz E, Bischof RH, Russmayer H, Ferrero P, Peacock S, Sauer M (2021). Efficient conversion of hemicellulose sugars from spent sulfite liquor into optically pure l-lactic acid by *Enterococcus mundtii*. Bioresour Technol.

[51] Wang A, Gao L, Ren N, Xu J, Liu C (2009). Bio-hydrogen production from cellulose by sequential co-culture of cellulosic hydrogen bacteria of *Enterococcus gallinarum* G1 and *Ethanoigenens harbinense* B49. Biotechnol Lett.

[52] Li J, Yuan X, Desta ST, Dong Z, Mugabe W, Shao T (2018). Characterization of *Enterococcus faecalis* JF85 and *Enterococcus faecium* Y83 isolated from Tibetan yak (*Bos grunniens*) for ensiling *Pennisetum sinese*. Bioresour Technol.

[53] de Almeida CV, Taddei A, Amedei A (2018). The controversial role of *Enterococcus faecalis* in colorectal cancer. Ther Adv Gastroenter.

[54] Franz CMAP, Huch M, Abriouel H, Holzapfel W, Gálvez A (2011). Enterococci as probiotics and their implications in food safety. Int J Food Microbiol.

[55] Sun X, Yang Y, Zhang N, Shen Y, Ni J (2015). Draft genome sequence of *Dysgonomonas macrotermitis* strain JCM 19375^T^, isolated from the gut of a termite. Genome Announc.

[56] Luo C, Li Y, Chen Y, Fu C, Long W, Xiao X (2019). Bamboo lignocellulose degradation by gut symbiotic microbiota of the bamboo snout beetle *Cyrtotrachelus buqueti*. Biotechnol Biofuels.

[57] Bridges CM, Gage DJ (2021). Development and application of aerobic, chemically defined media for *Dysgonomonas*. Anaerobe.

[58] Zheng J, Wittouck S, Salvetti E, Franz CMAP, Harris HMB, Mattarelli P (2020). A taxonomic note on the genus *Lactobacillus*: Description of 23 novel genera, emended description of the genus *Lactobacillus* Beijerinck 1901, and union of *Lactobacillaceae* and *Leuconostocaceae*. Int J Syst Evol Micr.

[59] Ong RG, Higbee A, Bottoms S, Dickinson Q, Xie D, Smith SA (2016). Inhibition of microbial biofuel production in drought-stressed switchgrass hydrolysate. Biotechnol Biofuels.

[60] Zhang M, Chou Y-C, Howe W, Eddy C, Evans K, Mohagheghi A. *Zymomonas* pentose-sugar fermenting strains and uses thereof. United States Patent US7223575B; 2007.

[61] Eaton AD, American Public Health Association, American Water Works Association, Water Environment Federation. Standard Methods for the Examination of Water and Wastewater. 2005.

[62] Foster CE, Martin TM, Pauly M (2010). Comprehensive compositional analysis of plant cell walls (lignocellulosic biomass) Part II: carbohydrates. JOVE-J Vis Exp.

[63] Sluiter A, Hames B, Ruiz R, Scarlata C, Sluiter J, Templeton D, et al. Determination of Structural Carbohydrates and Lignin in Biomass. 2008.

[64] Schwalbach MS, Keating DH, Tremaine M, Marner WD, Zhang Y, Bothfeld W (2012). Complex physiology and compound stress responses during fermentation of alkali-pretreated corn stover hydrolysate by an *Escherichia coli* ethanologen. Appl Environ Microb.

[65] Price MN, Dehal PS, Arkin AP (2010). FastTree 2—approximately maximum-likelihood trees for large alignments. PLoS ONE.

[66] Castresana J (2000). Selection of conserved blocks from multiple alignments for their use in phylogenetic analysis. Mol Biol Evol.

[67] Talavera G, Castresana J (2007). Improvement of phylogenies after removing divergent and ambiguously aligned blocks from protein sequence alignments. Syst Biol.

[68] Kumar S, Stecher G, Li M, Knyaz C, Tamura K (2018). MEGA X: molecular evolutionary genetics analysis across computing platforms. Mol Biol Evol.

[69] Klindworth A, Pruesse E, Schweer T, Peplies J, Quast C, Horn M (2013). Evaluation of general 16S ribosomal RNA gene PCR primers for classical and next-generation sequencing-based diversity studies. Nucleic Acids Res.

[70] Caporaso JG, Kuczynski J, Stombaugh J, Bittinger K, Bushman FD, Costello EK (2010). QIIME allows analysis of high-throughput community sequencing data. Nat Methods.

[71] Fortney NW, Hanson NJ, Rosa PRF, Donohue TJ, Noguera DR (2021). Diverse profile of fermentation byproducts from thin stillage. Front Bioeng Biotechnol.

