# Supplementary material for: Utilization of lignocellulosic biofuel conversion residue by diverse microorganisms
Source: Biotechnol Biofuels Bioprod. 2022 Jun 24;15:70. doi: 10.1186/s13068-022-02168-0 (PMC9233362; doi:10.1186/s13068-022-02168-0)
Supplement: Supplementary file 1 — Additional file 1: Figures S1. and S2. and Tables S1, S3 and S4 [file 13068_2022_2168_MOESM1_ESM.docx]

**Table S1.** Lignocellulosic conversion residue (LCR) composition

| Component | g/L | g COD/L |
| --- | --- | --- |
| Uncharacterized soluble |  | 0.71 ± 1.67 |
| Insoluble | 2.23 ± 1.16 | 3.35 ±1.74 |
| Lignols | 0.65 ± 0.06 | 1.15 ± 0.11 |
| Acetamide | 3.78 ± 0.53 | 4.09 ± 0.57 |
| Protein | 1.37 ± 0.65 | 2.06 ± 0.98 |
| Ribose | 0.66 ± 0.02 | 0.70 ± 0.02 |
| Amino Acids | 0.35 ± 0.00 | 0.51 ± 0.01 |
| Glycerol | 0.42 ± 0.10 | 0.51 ± 0.13 |
| Xylitol | 1.16 ± 0.48 | 1.34 ± 0.55 |
|  |  |  |
| C1-C4 metabolites |  |  |
| Formate | 0.45 ± 0.01 | 0.16 ± 0.00 |
| Acetate | 3.23 ± 0.06 | 3.44 ± 0.07 |
| Pyruvate | 1.75 ± 0.22 | 1.59 ± 0.20 |
| Succinate | 0.46 ± 0.19 | 0.44 ± 0.18 |
| Ethanol | 0.29 ± 0.12 | 0.60 ± 0.24 |
|  |  |  |
| Monomeric carbohydrates |  |  |
| Arabinose | 4.10 ± 0.14 | 4.37 ± 0.15 |
| Fucose | 0.08 ± 0.00 | 0.10 ± 0.00 |
| Galactose | 0.35 ± 0.04 | 0.37 ± 0.04 |
| Glucose | 0.55 ± 0.05 | 0.58 ± 0.06 |
| Mannose | 0.41 ± 0.04 | 0.44 ±0.04 |
| Rhamnose | 0.07 ± 0.00 | 0.09 ± 0.00 |
| Xylose | 3.26 ± 0.51 | 3.47 ± 0.54 |
|  |  |  |
| Oligomeric carbohydrates |  |  |
| Arabinose | 1.90 ± 0.22 | 2.02 ± 0.23 |
| Cellobiose | 0.89 ±0.78 | 1.00 ±0.88 |
| Cellulose | 17.49 ± 2.84 | 20.71 ± 3.36 |
| Fucose | 0.07 ±0.00 | 0.09 ±0.00 |
| Galactose | 1.28 ± 0.04 | 1.36 ± 0.04 |
| Glucose | 2.51 ± 0.23 | 2.68 ± 0.24 |
| Mannose | 0.72 ± 0.09 | 0.77 ± 0.10 |
| Rhamnose | 0.29 ± 0.01 | 0.37 ± 0.01 |
| Xylose | 5.40 ± 0.90 | 5.75 ± 0.96 |
|  |  |  |
| Total | 56.2 ± 2.3 | 64.1 ± 2.7 |


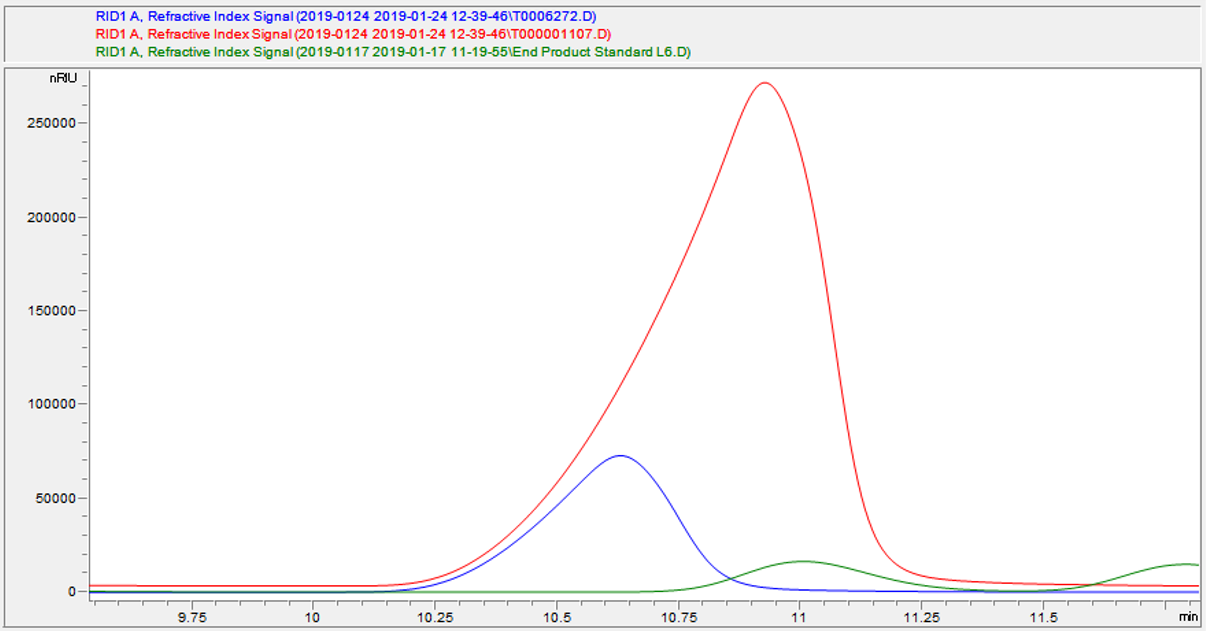


**Fig. S1.** HPLC chromatogram showing the coelution of the glucose analytical standard (green line) and an unidentified metabolite at high concentration (red line), but a shorter retention time when the metabolite is at a lower concentration (blue line).

**Table S3.** Synthetic Conversion Residue (SynCR) composition

|  |  |  |
| --- | --- | --- |
| Major components | g/L | g COD/L |
| Sodium acetate | 4.585 | 4.89 |
| Ethanol | 0.200 | 0.42 |
| Sodium formate | 0.900 | 0.31 |
| Cellobiose | 0.200 | 0.22 |
| Glucose | 0.400 | 0.43 |
| Glycerol | 0.400 | 0.49 |
| Sodium pyruvate | 1.645 | 1.61 |
| Sodium succinate | 0.600 | 0.57 |
| Xylitol | 1.400 | 1.62 |
| Xylose | 8.000 | 8.53 |
| Arabinose | 6.000 | 6.40 |
| Galactose | 1.500 | 1.60 |
| Fructose | 2.340 | 2.96 |
| Acetamide | 3.780 | 4.09 |
| Sigmacell 50 | 24.660 | 29.20 |
| KH2PO4 | 2.413 |  |
| CaCl2•6H2O | 0.294 |  |
| MgCl2•6H2O | 2.584 |  |
| MgSO4•7H2O | 0.861 |  |
| NaCl | 2.342 |  |
| NH4Cl | 1.779 |  |
| KCl | 0.823 |  |
|  |  |  |
| Micronutrients | mg/L |  |
| FeSO4•7H2O | 2.0 |  |
| H3BO3 | 15.8 |  |
| CuSO4•5H2O | 0.1 |  |
| Na2MoO4 | 0.1 |  |
| MnCl2•4H2O | 13.8 |  |
| ZnSO4•7H2O | 2.8 |  |
|  |  |  |
| Amino acids | mg/L | g COD/L |
| Alanine | 32.2 |  |
| Arginine | 18.5 |  |
| Asparagine | 5.3 |  |
| Aspartic acid | 8.6 |  |
| Cysteine | 18.2 |  |
| Glutamine | 11.2 |  |
| Glutamic acid | 4.8 |  |
| Glycine | 11.7 |  |
| Histidine | 2.4 |  |
| Isoleucine | 16.6 |  |
| Leucine | 23.1 |  |
| Lysine | 14.0 |  |
| Methionine | 22.4 |  |
| Phenylalanine | 58.1 |  |
| Proline | 18.6 |  |
| Serine | 10.9 |  |
| Threonine | 11.9 |  |
| Tryptophan | 30.6 |  |
| Valine | 18.2 |  |
| Tyrosine | 82.7 |  |
| *Sum* |  | 0.51 |
|  |  |  |
| Lignocellulose-derived inhibitors (LDIs) | mg/L | g COD/L |
| Coumaroyl amide | 166.0 |  |
| Feruloyl amide | 132.0 |  |
| Syringamide | 7.0 |  |
| Benzoic acid | 46.0 |  |
| Coumaric acid | 20.0 |  |
| 4-Hydroxybenzoic acid | 9.0 |  |
| Ferulic acid | 6.0 |  |
| Syringic acid | 3.0 |  |
| Vanillic acid | 26.0 |  |
| Acetovanillone | 5.0 |  |
| Coniferyl alcohol | 19.0 |  |
| 4-Hydroxyacetophenone | 4.0 |  |
| *Sum* |  | 1.15 |

Table S4. Yeast SynCR Vitamin Dependency

| **Species** | **No additives** | **YNB** | **Biotin** | **Inositol** | **Pyridoxine/Nicotinic Acid** | **All 3** |
| --- | --- | --- | --- | --- | --- | --- |
| *Blastobotrys illinoiensis* | + | + | + | + | + | + |
| *Blastobotrys raffinosifermentans* | X | + | X | X | X | X |
| *Debaryomyces prosopidis* | X | + | + | X | X | + |
| *Blastobotrys capitulata* | X | + | N/A | N/A | N/A | N/A |
| *Wickerhamiella dulcicola* | X | + | N/A | N/A | N/A | N/A |
| *Blastobotrys terrestris* | X | + | N/A | N/A | N/A | N/A |
| *Schwanniomyces pseudopolymorphus* | X | + | N/A | N/A | N/A | N/A |

"+” indicates growth, X indicates no growth, N/A was not tested.


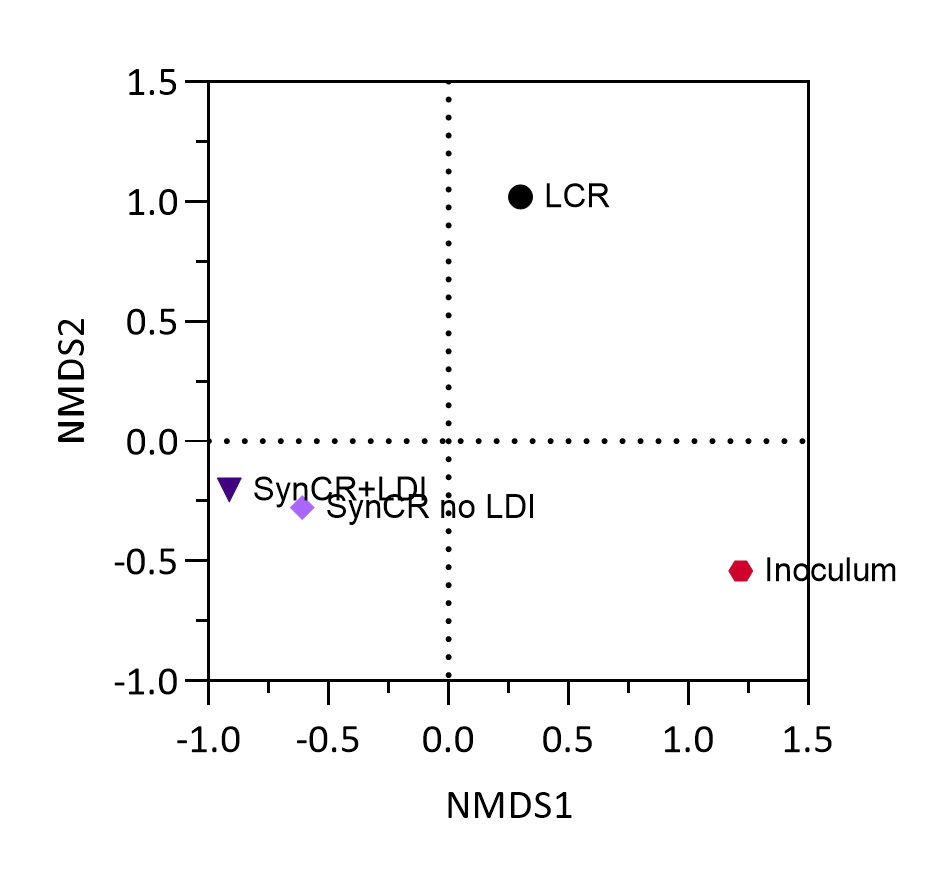


**Fig S2.** Non-metric multidimensional scaling plot showing the relationship between the microbial communities following a 1-week growth period on different types of conversion residues.
